# Chronic Intermittent Hypoxia Differentially Impacts Different States of Inspiratory Activity at the Level of the preBötzinger Complex

**DOI:** 10.3389/fphys.2017.00571

**Published:** 2017-08-28

**Authors:** Alfredo J. Garcia, Tatiana Dashevskiy, Maggie A. Khuu, Jan-Marino Ramirez

**Affiliations:** ^1^Institute for Integrative Physiology, The University of Chicago Chicago, IL, United States; ^2^Department of Medicine, Section of Emergency Medicine, The University of Chicago Chicago, IL, United States; ^3^Center for Integrative Brain Research, Seattle Children's Research Institute Seattle, WA, United States; ^4^Departments of Neurological Surgery and Pediatrics, University of Washington Seattle, WA, United States

**Keywords:** chronic intermittent hypoxia, preBötzinger complex, hypoxia, brain, sleep apnea syndromes, rhythmicity

## Abstract

The preBötzinger complex (preBötC) is a medullary brainstem network crucially involved in the generation of different inspiratory rhythms. In the isolated brainstem slice, the preBötC reconfigures to produce different rhythms that we refer to as “fictive eupnea” under baseline conditions (i.e., carbogen), and “fictive gasping” in hypoxia. We recently demonstrated that fictive eupnea is irregular following exposure to chronic intermittent hypoxia (CIH). However, it is unknown how CIH impacts fictive gasping. To address this, brain slices containing the preBötC were prepared from control and CIH exposed mice. Electrophysiological recordings of rhythmogenesis were obtained during the perihypoxic interval. We examined how CIH affects various dynamic aspects of the rhythm characterized by: (1) the irregularity score (IrS), to assess burst-to-variability; (2) the fluctuation value (χ), to quantify the gain of oscillations throughout the time series; and (3) Sample Entropy (sENT), to characterize the pattern/structure of oscillations in the time series. In baseline conditions, CIH increased IrS of amplitude (0.21 ± 0.2) and χ of amplitude (0.34 ± 0.02) but did not affect sENT of amplitude. This indicated that CIH increased burst-to-burst irregularity and the gain of amplitude fluctuations but did not affect the overall pattern/structure of amplitude oscillations. During the transition to hypoxia, 33% of control rhythms whereas 64% of CIH-exposed rhythms showed no doubling of period, suggesting that the probability for stable rhythmogenesis during the transition to hypoxia was greater following CIH. While 29% of control rhythms maintained rhythmicity throughout hypoxia, all slices from CIH exposed mice exhibited rhythms throughout the hypoxic interval. During hypoxia, differences in χ for amplitude were no longer observed between groups. To test the contribution of the persistent sodium current, we examined how riluzole influenced rhythmogenesis following CIH. In networks exposed to CIH, riluzole reduced the IrS of amplitude (-24 ± 14%) yet increased IrS of period (+49 ± 17%). Our data indicate that CIH affects the preBötC, in a manner dependent on the state of the oxygenation. Along with known changes that CIH has on peripheral sensory organs, the effects of CIH on the preBötC may have important implications for sleep apnea, a condition characterized by rapid transitions between normoxia and hypoxia.

## Introduction

Neuronal networks are very sensitive to the state of oxygenation and are capable of responding to hypoxia and reoxygenation, which can induce various forms of plasticity (Blitz and Ramirez, 2002; Peng et al., 2009; Garcia et al., 2010; Nichols et al., 2012; Quintana et al., 2015; Devinney et al., 2016). These forms of plasticity can be adaptive or detrimental depending on the specific patterns of induction (Navarrete-Opazo and Mitchell, 2014; Quintana et al., 2015). Chronic intermittent hypoxia (CIH) is one particular pattern of hypoxia, which has been associated with several clinical conditions. CIH can persist for several years or throughout a lifetime. It can be caused e.g., by irregular breathing and apneas that are found in prematurity (Di Fiore et al., 2016). Beyond the perinatal age, CIH caused by sleep apnea (SA), which can present either as obstructive or central apnea (Ramirez et al., 2013). Chronic conditions of IH can have detrimental consequences on the quality of life by disrupting restful sleep, impairing cognitive function (Gozal et al., 2010; Davies and Harrington, 2016), promoting respiratory dysfunction (Malhotra and White, 2002; Dempsey et al., 2010; Ramirez et al., 2013), and increasing the risk for cardiovascular disease (Javaheri et al., 2017). Human studies also suggest that the etiology of SA is rooted in changes to cardio-respiratory control (Deacon and Catcheside, 2015). Animal studies using CIH as a model of SA also support the view that SA changes respiratory control (Chopra et al., 2016).

We have recently reported that CIH impacts respiratory activity generated centrally within the preBötzinger complex (preBötC). CIH increases baseline burst-to-burst fluctuations of rhythm generation, as defined by increases in the irregularity score. These changes were accompanied by intermittent transmission of the premotor rhythm to the motor pool, a finding which may have important implications for the etiology of obstructive SA (Garcia et al., 2016).

It has been demonstrated that the preBötC can reconfigure in response to hypoxia and is thus capable of contributing to several rhythms germane to respiration, in particular eupneic inspiratory activity, gasping, sighing, and post-hypoxic recovery (Lieske et al., 2000; Garcia et al., 2013). Each of these types of inspiratory activities is associated with distinct cellular and network properties within the preBötC (Lieske et al., 2000; Pena et al., 2004; Lieske and Ramirez, 2006a,b; Tryba et al., 2008; Hill et al., 2011). This raises the question whether the different states of the preBötC are affected in a similar or in a differential manner by CIH exposure. This question is relevant for understanding conditions such as sleep apnea, in which patients frequently transition between normoxic and hypoxic conditions. The objective of this study was to examine how CIH affects the state of the inspiratory rhythm generating network during changes in oxygenation (i.e., the generation of fictive eupnea and fictive gasping). Electrophysiological studies were conducted in the preBötC of brainstem slices harvested from either control or CIH-exposed mice. These studies demonstrate that CIH differentially altered the irregularity score and the sample entropy of rhythm generation. The stability of rhythmogenesis following CIH was dependent on the state of oxygenation—after CIH the isolated preBötC appears to be less sensitive to hypoxia. These effects were accompanied by differences in sensitivity to riluzole, a pharmacological agent known to block fictive gasping presumably by inhibiting the persistent sodium current, I_NaP_ (Del Negro et al., 2002; Pena et al., 2004; Rybak et al., 2007). These findings may be fundamentally important for understanding how conditions such as sleep disordered breathing and apneas of prematurity affect the ventilatory response, which involves changes in both the peripheral and central components of the respiratory system.

## Methods

### Ethics statement

Experiments were conducted using CD1 mice and protocols were approved by the Animal Care and Use Committee at Seattle Children's Research Institute and at The University of Chicago in accordance with the National Institutes of Health guidelines. All animal subjects were housed at 21°C and in a 12/12 h light cycle where the light phase was from 07:00 to 19:00. Animals had access to food and water *ad libitum*.

### Exposure to CIH

Neonatal mice (beginning from postnatal day 0–2) and their dam were exposed to CIH. CIH occurred during the light cycle and lasting for 8 h/day (i.e., 80 intermittent hypoxia cycles/day), for 10 consecutive days. Litters were culled down to a total of eight pups prior to CIH exposure.

As described previously (Garcia et al., 2016) a single hypoxic bout was achieved by flowing 100% N_2_ into the chamber for ~60 s created a hypoxic state where the nadir O_2_ value reached 4–5% O_2_ (for 5–7 s). The hypoxic bout was followed by a 300 s air break achieved by flushing with air (~21% O_2_). The return to air restored a normoxic state (18–20%) within 60 s following hypoxia. Environmental CO_2_ did not rise >0.02% during any phase. Both pups and their dam were exposed to the CIH paradigm.

### Brain slice preparation and electrophysiological recordings

Transverse brainstem slices containing the preBötC were prepared from either CIH-exposed mice (12–48 h after the end of the CIH paradigm) or naïve (i.e., control) CD1 mice (postnatal day 10–12). Mice were rapidly decapitated or anesthetized with isofluorane and then decapitated at which point the brainstem was removed and prepared for slicing. The isolated brainstem was glued to an agar block (dorsal face to agar) with the rostral face up toward the vibratome blade and submerged in artificial cerebrospinal fluid (aCSF, ~4°C) equilibrated with Carbogen (95% O_2_–5% CO_2_). One hundred to two hundred micrometer serial transverse slices at a 20° angle were made in a rostral to caudal direction until disappearance of the parafacial group and appearance of the inferior olive, nucleus ambiguus, the hypoglossal nucleus, and the opening of the fourth ventricle as also described previously by others (Ballanyi and Ruangkittisakul, 2009). A 550 μm thick rhythmic slice containing the preBötC was made. Our approach encompasses the preBötC as we have previously documented (Viemari et al., 2013). A preBötC network in the brainstem slices was considered intact if transmission of the preBötC rhythm to the hypoglossal pool was consistent (i.e., >40% of preBötC bursts transmitted to the motor pool) and/or a synchronous preBötC rhythm was detected bilaterally in each preBötC prior to the start of our recordings.

The composition of artificial cerebral spinal fluid (aCSF) was (in mM): 118 NaCl, 3.0 KCl, 25 NaHCO_3_, 1 NaH_2_PO_4_, 1.0 MgCl_2_, 1.5 CaCl_2_, 30 D-glucose. The aCSF had an osmolarity of 308 ± 2 mOSM and a pH of 7.40 to 7.45 when equilibrated with gas mixtures containing 5% CO_2_ at ambient pressure. Rhythmic activity from the preBötC was induced by raising extracellular KCl to 8.0 mM. Baseline conditions were made by equilibrating aCSF with carbogen (95% O_2_, 5% CO_2_) while hypoxic conditions were made by aerating with 95% N_2_, 5% CO_2_. Exposure to hypoxia lasted for 600 s. Despite the equilibration of aCSF with 0% O_2_, hypoxic media contained some O_2_ (Garcia et al., 2010; Hill et al., 2011).

Extracellular population activity was recorded with glass suction pipettes (tip resistance <1 MΩ) filled with aCSF, and were positioned over the ventral respiratory column containing the preBötC. Signals were amplified 10,000X, filtered (low pass, 1.5 kHz; high pass, 250 Hz), rectified, and integrated using an electronic filter. Extracellular recordings were acquired in pCLAMP software (Molecular Devices, Sunnyvale, CA) and were analyzed *post-hoc* in Clampfit software (version 10.2). In some cases, population recordings from two preBötC brainstem slices were recorded simultaneously within a single recording chamber. In situations where two slices were used, the slices were positioned in a staggered arrangement such that media flow was not obstructed for either preparation.

### Plethysmography

Whole animal plethysmography was performed under unrestrained conditions in mice (P10 to P13) using a commercial environmental chamber (Buxco Research System). Briefly, normoxic/normocapnic air (19 to 21% O_2_, 0% CO_2_) was replaced by hypoxic breathing gas (10% O_2_, 0% CO_2_) for 5 min. We measured respiratory frequency (breath per min), tidal volume (μL per breath), and minute ventilation (μL^.^g^−1.^min^−1^). Measurements were performed during (a) control period under air, prior to hypoxia, (b) the second minute of hypoxia, (c) the fifth minute of hypoxia, (d) the post-hypoxic period (2 and 4 min following the end of hypoxia when normoxia was restored in the chamber), and (e) 10 min later. Mice were kept thermoneutral at 33°C throughout the plethysmography session.

### Analysis and statistics

**Figure 1:**
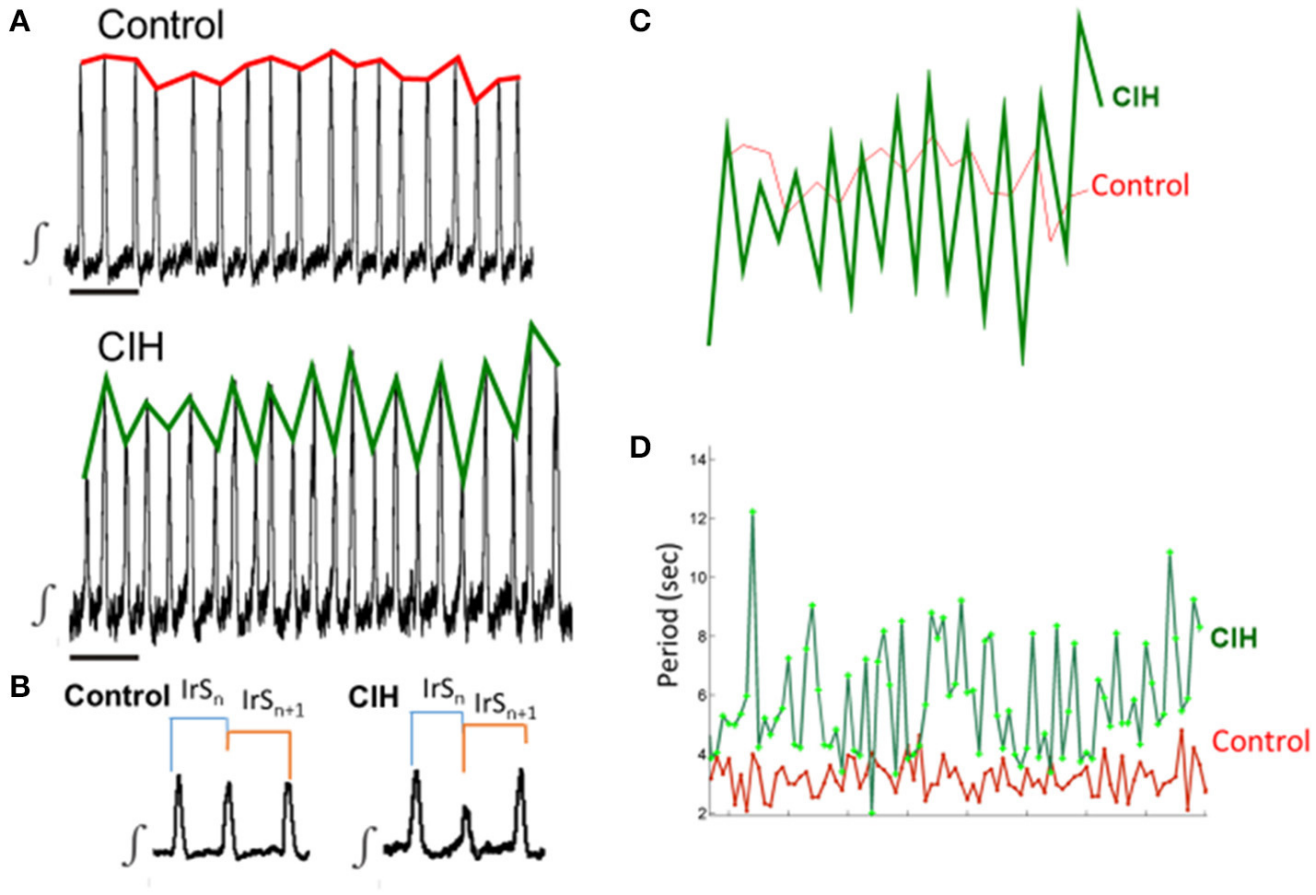
Fluctuations in network parameters can be detected and characterized by different methods. **(A)** Representative examples of integrated traces of preBötC activity from the control (top) and CIH (bottom) groups. Red line highlights the periodic fluctuation in burst amplitude throughout the control rhythm. Green line highlights the periodic fluctuation in burst amplitude throughout the time-series. Scale bar: 10 s. **(B)** Expanded traces of representative rhythms from **(A)** group illustrating the time window for the calculation of irregularity score (IrS) for the *n*^th^ and the n^th^+1 cycles (i.e., the burst to burst window) for both control and CIH rhythms. **(C)** Overlay of the fluctuation of burst amplitudes for the control rhythm in **(A)** (red) and the CIH rhythm in **(B)** (green) demonstrating the presence of fluctuations in both rhythms yet larger in magnitude in the CIH group. The gain of fluctuations can be described by the fluctuation value (χ) see methods for formula. **(D)** Example of the fluctuation of period for a control rhythm (red) and a CIH rhythm (green). Representative integrated traces showing the fluctuations in period can be observed in Figure 2, 4.

To resolve how CIH affects the dynamical system properties of rhythm generation we examined (1) burst-to-burst variability using the irregularity score (IrS; Zanella et al., 2014); (2) the magnitude of fluctuation in the network rhythm was defined by its fluctuation value (χ); and (3) the predictability and thus, the complexity of the network rhythm throughout the time series using sample entropy (sENT; Richman and Moorman, 2000). For further detail see Figure 1.

To calculate IrS of period (IrS_P_) for a single cycle we used the following formula (Figure 1B; see also Telgkamp et al., 2002): IrS_P − *N*_ = 100 × ABS (*P*_*N*_ - *P*_*N* − 1_)/*P*_*N* − 1_, where IrS_P− *N*_ is the IrS_P_ of the *N*th cycle, *P*_*N*_ is its period, *P*_*N* − 1_ is the period of the preceding cycle, and ABS is the absolute value. We also apply this formula to assess the irregularity of burst amplitude IrS_AMP_ by replacing in the previous formula the period by the amplitude of the integrated and rectified inspiratory burst (with unchanged time constants throughout the study). The mean irregularity score for all cycles was calculated for each rhythmic activity generated by a given slice.

The network population rhythm is the result of phase-locking activity between weakly coupled rhythmically active neurons, which includes bursting pacemakers and non-pacemaker neurons (Pena et al., 2004; Carroll et al., 2013). Changes in intrinsic neuronal and/or synaptic properties by CIH have the potential to alter the inherent fluctuations of the network rhythm (i.e., periodic fluctuations in the amplitude and/or period (illustrated with red or green lines that follow the peak amplitudes in Figure 1A).

If CIH caused changes to the periodic fluctuations of amplitude and period that span across more than two cycles in the time-series (Figure 1A), the burst-to-burst window used to calculate the irregularity score (Figure 1B) would provide limited insights into the phenomenon. To address this limitation of irregularity score, we calculated the fluctuation value (χ) and sample entropy (sENT) of metrics in the time series of each rhythm. While sENT is a modification of approximate entropy used for characterizing the complexity of physiological time-series signals (Richman and Moorman, 2000) that describes the frequency of repetitive fluctuations in the time series, we introduce χ to characterize the gain of these repetitive fluctuations in the time series (see Figures 1C,D).

To calculate the fluctuations, a Butterworth filter was applied to event values plotted sequentially and local minima and maxima were detected. The fluctuation value (χ) for a given metric was calculated as the difference between local maxima and minima, χ_i_, where i is single value of fluctuation and n- total number of fluctuations in an 300–400 s window. The average fluctuation was calculated as $\frac{1}{n}\overset{n}{\underset{i=1}{\sum }}x_{i}$. Thus, χ_i_ was used to identify whether the magnitude of fluctuations in a given metric of rhythm generation changed over time. To calculate χ of amplitude, amplitude events were normalized to its average. sENT was used to identify whether the overall predictability in the time series as given metric of rhythm generation changed over time. sENT was calculated as $-\log\frac{Nx}{Ny}$ where *N*_*x*_ and *Ny* are the number of pairs of dimension 3 and 2 with distance less than two standard deviations of a given time series data set. Thus, a lower value of sENT corresponded to a time series containing repetitive patterns (i.e., less structural predictability) and thus, complexity in the time-series. Both χ and sENT were calculated from a 300 to 400 s window. In some cases, too few events were available to calculate either χ or sENT for a given parameter and were not included in the respective analyses (see Table 1 for *n*-values).

**Table 1:** The fluctuation value (χ) and sample entropy (sENT) for period and amplitude during baseline, hypoxia, and in riluzole.

		**χ Baseline^1^**	**sENT Baseline^1^**	**χ During Hypoxia^2^**	**sENT during Hypoxia^2^**	**χ During Riluzole**	**sENT during Riluzole**
Period	Control (*n*)^3^	0.36 ± 0.02 (*n* = 31/31)	2.03 ± 0.10 (*n* = 30/31)	0.41 ± 0.03 (*n* = 17/27)	1.92 ± 0.10 (*n* = 15/ 27)	0.34 ± 0.02 (*n* = 9/ 9)	2.35 ± 0.09 (*n* = 9/9)
	CIH (*n*)^3^	0.44 ± 0.02 (*n* = 37/39)	2.05 ± 0.11 (*n* = 31/39)	0.36 ± 0.03 (*n* = 20/22)	1.87 ± 0.11 (*n* = 19/22)	0.43 ± 0.04 (*n* = 16/16)	1.97 ± 0.12 (*n* = 12/16)
	*P*-value	0.001	0.91	0.22	0.75	0.04	0.02
Amplitude	Control (*n*)^3^	0.27 ± 0.02 (*n* = 31/31)	1.66 ± 0.08 (*n* = 31/39)	0.43 ± 0.03 (*n* = 17/27)	1.96 ± 0.12 (*n* = 15/27)	0.31 ± 0.03 (*n* = 9/9)	2.06 ± 0.12 (*n* = 9/9)
	CIH (*n*)^3^	0.34 ± 0.02 (*n* = 39/39)	1.69 ± 0.09 (*n* = 34/39)	0.43 ± 0.09 (*n* = 20/22)	2.59 ± 0.11 (*n* = 19/22)	0.37 ± 0.04 (*n* = 16/16)	1.64 ± 0.14 (*n* = 16 /16)
	*P*-value	*P* = 0.006	0.80	0.94	0.54	0.30	0.04

1 *Baseline defined as being in carbogen*.

2 *Hypoxia defined as the final 400 s of hypoxic exposure*.

3 *n is represented as a fraction. The numerator is the n the rhythms for which each metric that was calculated and the denominator is the total number of rhythms recorded in each condition*.

Analysis for hypoxic rhythm generation from the preBötC was divided into early hypoxia and steady-state hypoxia. Early hypoxia was defined as the initial 200 s interval beginning when O_2_ in the circulating aCSF began to drop due to a switch to the hypoxic gas mixture. Kaplan-Meier estimator functions were used to describe the probability of stable rhythmogenesis during the early hypoxia phase. The endpoint for this analysis was defined as the first occurrence of an interburst interval (i.e., cycle period) during hypoxic augmentation, which was at least two times greater than the mean period of rhythmogenesis prior to hypoxia. Rhythms not demonstrating the defined endpoint during hypoxic augmentation were censored. Differences between the Kaplan-Meier estimator functions were determined using the Gehan-Breslow-Wilcoxon test. Steady-state hypoxia was defined as the final 400 s interval of hypoxia (Hill et al., 2011; Garcia et al., 2013). During this interval the oxygen sensitive current measuring bath O_2_ was clearly reduced from the baseline, stable and asymptotic. To determine whether changes in the fictive gasping rhythm was uniquely impacted by CIH, we calculated the normalized values for a given metric using the following:

$$\begin{array}{l} X_{\text{normalized}}=\frac{100^{*}\left(X_{hypoxia}-X_{baseline}\right)}{X_{baseline}}\;\% \end{array}$$

where X_normalized_ represents the normalized value of metric “X” during hypoxia.

X_hypoxia_ is the value of the metric “X” and X_baseline_ is the value of metric “X” during baseline prior to hypoxia.

A 2-way ANOVA was used to assess differences in respiratory metrics during the peri-hypoxic interval in plethysmography experiments. Unless noted otherwise, comparisons between two groups were made using unpaired *t*-test. Differences between two means with similar variance was defined by a *P* < 0.05. An *F*-test for unequal variance was also performed and in cases where the *P*-value for the *F*-test was *P* < 0.05, we considered that differences existed between groups due to unequal variances. All statistical comparisons were made in Prism 5 (GraphPad Software).

## Results

### CIH affects network dynamics during baseline conditions

**Figure 2:**
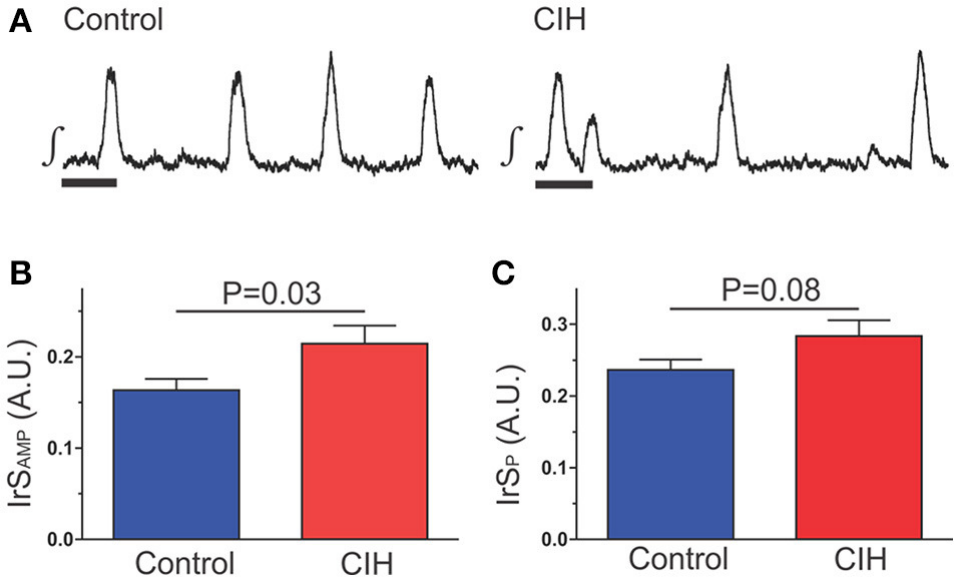
CIH increases the burst-to-burst irregularity of the isolated preBötC rhythm. **(A)** Representative examples of integrated traces of preBötC activity taken from control and CIH exposed mice. Scale Bar: 2 s **(B)** Comparison of the IrS_AMP_ of rhythmogenesis for control and CIH groups showing that following CIH IrS_AMP_ is increased. **(C)** Although the *P*-value for the comparison of IrS_P_ between control and CIH groups was >0.05, the *F*-ratio and its corresponding *P*-value indicated that CIH produced a large degree of variability in IrS_P_ and suggests that CIH indeed affects the burst-to-burst irregularity in period as previously reported (Garcia et al., 2016).

To identify how CIH affected rhythmogenesis during baseline conditions (i.e., in Carbogen), recorded rhythmogenesis from the isolated preBötC taken from either control mice (*n* = 31) or mice exposed to CIH (*n* = 39). We calculated the irregularity score (IrS), fluctuation value (χ), and sample entropy (sENT) for both burst amplitude and period. CIH affects the irregularity score of the network rhythms (Figure 2). The IrS of amplitude (IrS_AMP_) of control rhythms was 0.16 ± 0.01 (*n* = 31) while the IrS_AMP_ of following CIH was 0.21 ± 0.02 (*n* = 39). The IrS_AMP_ between the two groups was different from one another and exhibited unequal variances (Figure 2B; *P* = 0.03; F-ratio = 3.266, *P* = 0.001). Although, the IrS of period (IrS_P_) of control and CIH rhythms were similar (Figure 2C; control: 0.24 ± 0.01, *n* = 39 vs. CIH: 0.29 ± 0.02 *n* = 31; *P* = 0.08), an unequal variance existed between groups (F-ratio = 2.834; *P* = 0.004) suggesting that the effect CIH on the IrS_P_ was largely variable and different to the control group.

We next determined whether CIH affected the complexity of rhythmogenesis by calculating the fluctuation value, χ, and the entropy value, sEnt, during baseline conditions for both groups. The χ of period (χ_P_) and of the amplitude (χ_AMP_) were different between the two groups (Table 1). Neither sENT of amplitude (sENT_AMP_) nor sENT of period (sENT_P_) were different between the respiratory activity of controls vs. mice exposed to CIH (Table 1). Thus, CIH affects the dynamics of rhythm generation beyond burst-to-burst irregularity by increasing the gain of the fluctuations in rhythmogenesis, yet does not change predictability and complexity of the time series for these metrics.

### CIH affects the hypoxic response of the isolated respiratory network

**Figure 3:**
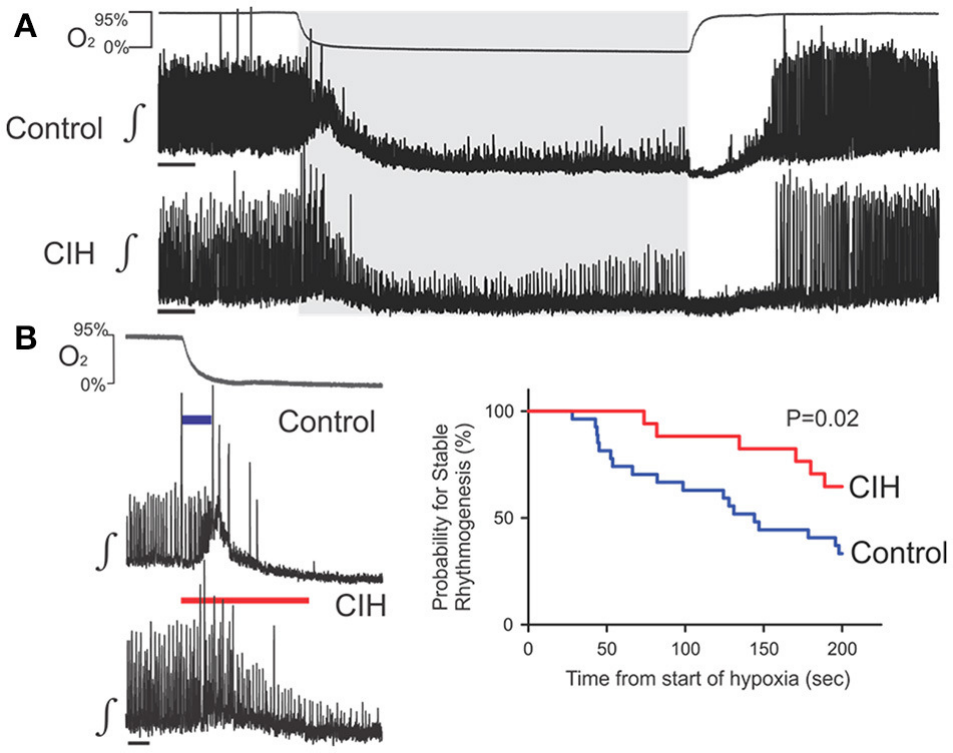
CIH affects the augmentation of rhythmogenesis during the initial period of hypoxia. **(A)** Representative examples of rhythmogenesis during the perihypoxic interval showing that hypoxia and reoxygenation produces similar stereotypical responses in the isolated preBötC from control and CIH exposed mice. **(B)** Left: Integrated traces of the preBötC rhythm (∫) during the initial period of hypoxia illustrating the early failure of rhythms from the control (top, blue bar: 44 s) vs. the CIH group (bottom, red bar 200 s). Scale Bars: 20 s. Right: Kaplan-Meier curves for the control (blue) and CIH (red) groups during the initial 200 s of hypoxia illustrating the increased propensity of failure in the control group compared to the CIH group.

**Figure 4:**
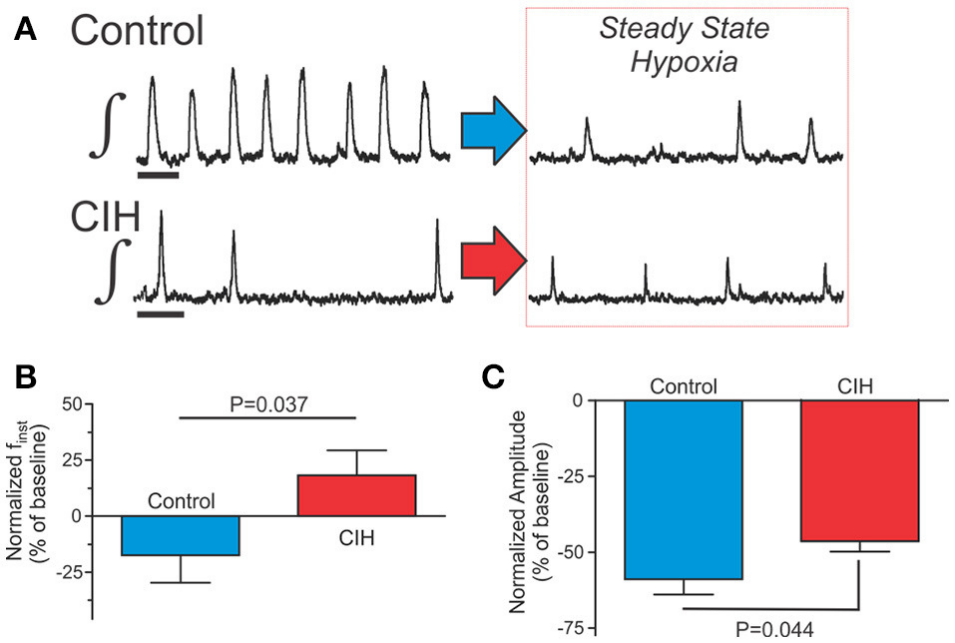
CIH affects rhythmogenesis later during hypoxia. **(A)** Representative examples of integrated traces of the preBötC rhythm from control (top) and CIH (bottom) in the final 400 s of a 600 s hypoxia. Scale Bar: 4 s **(B,C)** Comparisons showing that f_inst_ and IrS_AMP_ of hypoxic rhythmogenesis are different in the control vs. the CIH group.

To determine whether CIH affected the central respiratory network activity during hypoxia, we examined how the respiratory rhythmic activity in preBötC slices from control (*n* = 27) and CIH-exposed mice (*n* = 22) respond to hypoxia and reoxygenation. While control and CIH groups displayed qualitatively similar stereotypical responses to hypoxia and reoxygenation (Figure 3A), we found significant quantitative differences during the transition to and in hypoxia (Figure 3B) but not during post-hypoxic recovery (*data not shown*). During the transition to hypoxia (i.e., the first 200 s of exposure), 33% (*n* = 9 of 27) of control rhythms vs. 64% (*n* = 14 of 22) of CIH-exposed rhythms showed no doubling of period. Using the doubling of period as an endpoint, for Kaplan-Meier estimator functions revealed that exposure to CIH significantly increased the probability for stable rhythmogenesis (Figure 3B). Later during steady-state hypoxia (i.e., the final 400 s of hypoxia), 18% (*n* = 5 of 27) of control networks failed to generate respiratory rhythmic activity which contrasted the continued rhythmogenesis produced by all CIH-exposed inspiratory networks (*n* = 22; Figure 4A). When comparing the change between the control and CIH groups, normalized instantaneous frequency (f_inst_) during hypoxia was reduced by −17 ± 12% in the control group; whereas, in the CIH group in normalized f_inst_ during hypoxia increased by +18 ± 11% (Figure 4B). Normalized network burst amplitude during hypoxia was reduced by −59 ± 5% in the control group while it was only reduced by −46 ± 3% in the CIH group (Figure 4C) later during hypoxia. Interestingly, we observed an unequal variance between control and CIH exposed mice with regards to the normalized amplitude (F-ratio = 3.175, *P* = 0.01), IrS_AMP_ (F-ratio = 3.082, *P* = 0.013), and IrS_P_ (F-ratio = 3.175, *P* = 0.010) where the variance of the dataset during hypoxia was consistently larger in the control group vs. the CIH-exposed group. However, during hypoxia, the χ and sENT values from the CIH for both period and amplitude were not different from the respective control group (Table 1). These findings together indicate that CIH differentially affects rhythmogenesis in well-oxygenated states and during hypoxia, and suggests that the CIH-induced effects are state-dependent.

### Rilozule sensitivity of rhythmogenesis changes following CIH

**Figure 5:**
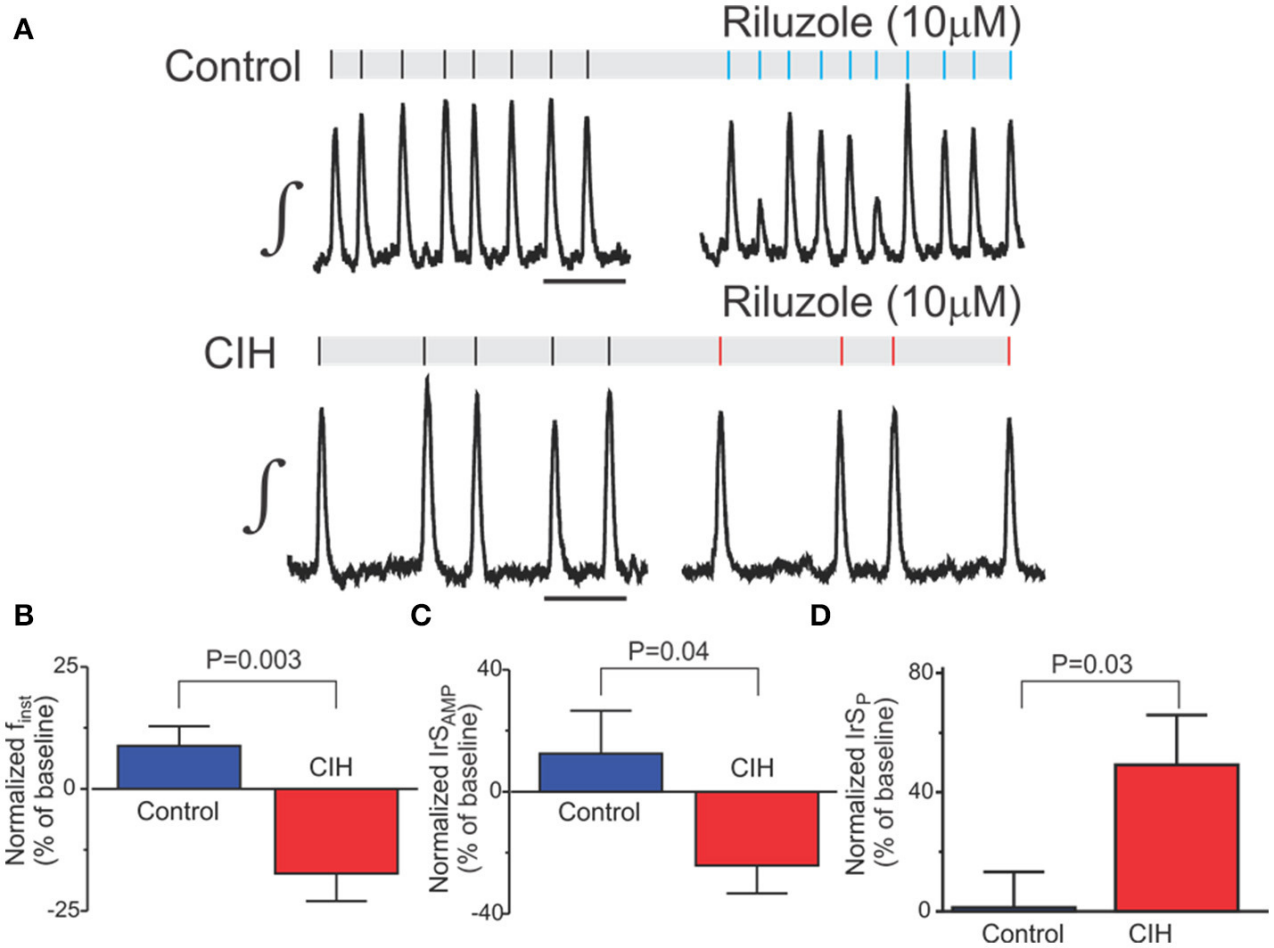
CIH changes riluzole sensitivity of rhythmogenesis. **(A)** Representative examples of integrated traces of the preBötC rhythm from control (top) and CIH (bottom) before and during exposure to riluzole. Scale Bar: 5 s **(B–D)** CIH affects the change in finst, IrS_AMP_, and IrS_P_ in riulzole.

Because of the differences during hypoxia, and the documented riluzole sensitivity of the preBötC during hypoxia (Pena et al., 2004), we sought to assess how CIH impacted riluzole sensitivity of the preBötC following CIH. Riluzole (10 μM) caused several observable changes in rhythmogenesis that were different in control (*n* = 9) vs. in mice that were exposed to CIH (*n* = 16; Figure 5A). In riluzole, f_inst_ was greater (*P* = 0.003) in control (0.40 ± 0.04) vs. networks from mice exposed to CIH (0.21 ± 0.03). The differential effect of riluzole on f_inst_ was also reflected by the greater reduction in the change of f_inst_ from the CIH group (Figure 5B). Differences were also evident in the change of IrS_AMP_ between groups. Specifically, the change in IrS_AMP_ did not exceed 100% in the control group when exposed to riluzole. Yet, in the CIH group, riluzole increased IrS_AMP_ by >100% in 3 of 16 recordings of respiratory rhythmic activity. When comparing these groups, the mean effect of riluzole was a decrease in IrS_AMP_ by -24 ± 14% (Figure 5C, *P* = 0.04) and an increase in IrS_P_ +49 ± 17% (Figure 5D, *P* = 0.030) for the CIH exposed group. Although, riluzole did not produce differences in χ_AMP_ between control and CIH, the riluzole exposure increased χ_P_ of rhythmogenesis following the CIH (Table 1). These findings suggest that CIH enhances a riluzole sensitive mechanism to reduces both burst-to-burst variability and the gain of fluctuations in rhythmogenesis. Interestingly, riluzole reduced both sENT_P_ and sENT_AMP_ in the CIH exposed group (Table 1) and suggests that networks exposed to CIH possess a riluzole sensitive mechanism, which decreases the degree of overall predictability of rhythmogenesis. Thus, together these observations support the notion that CIH fundamentally changes rhythmogenesis from the preBötC.

### CIH augments respiratory frequency *in vivo*

**Figure 6:**
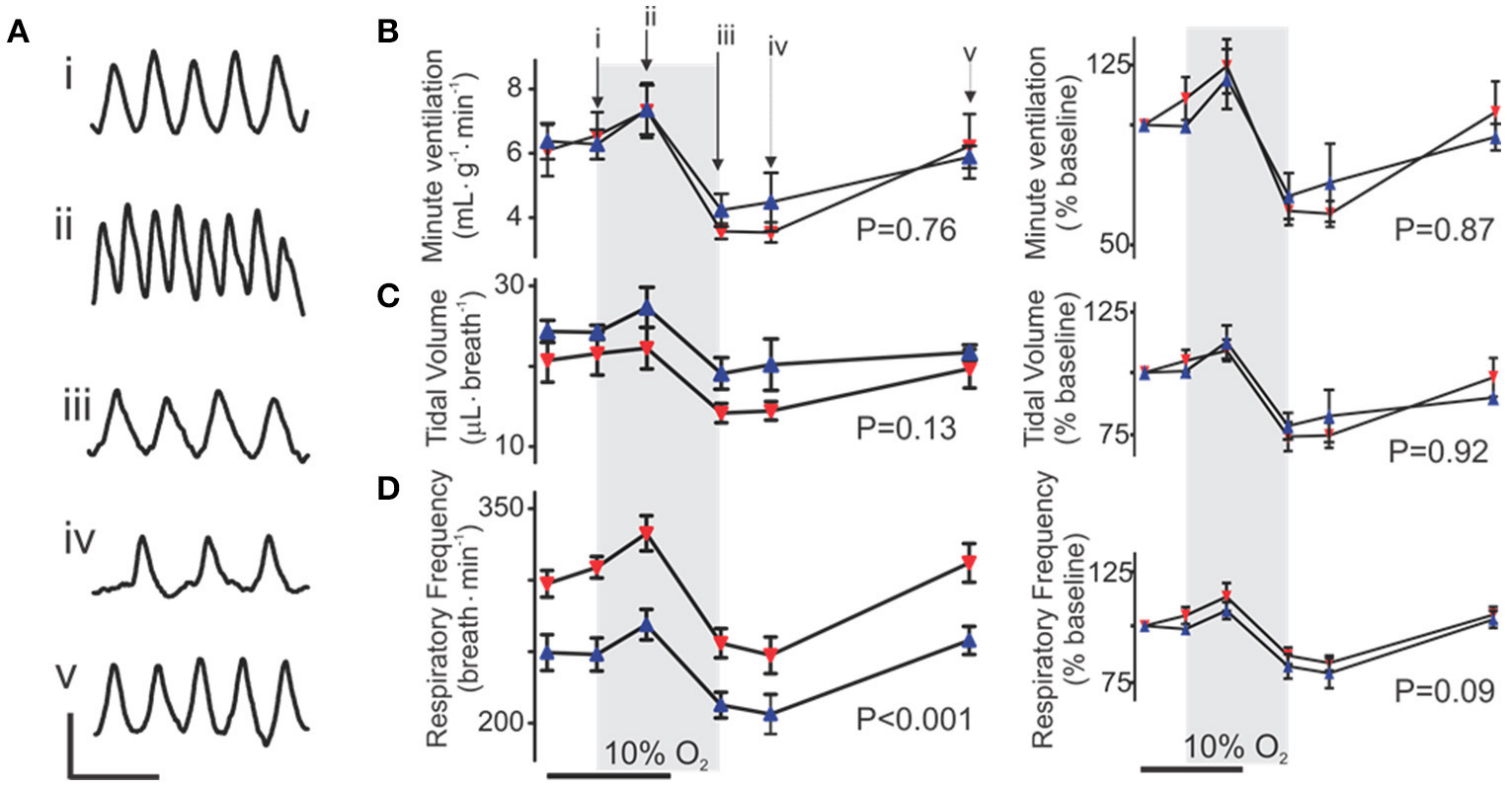
Following CIH respiratory frequency is increased *in vivo*. **(A)** Representative plethysmography traces from a control mouse illustrating the changes to frequency during the peri-hypoxic interval. Scale Bars: 500 ms (horizontal); 20 μL (vertical) Comparison of **(B)** Minute Ventilation, **(C)** Tidal Volume **(D)** Respiratory Frequency between control and CIH exposed mice. In **(B–D)** left shows absolute values, right shows normalized values. Each metric was normalized to each respective baseline metric while breathing air 2 min before hypoxic exposure.

To access how changes caused by CIH affect the ventilatory response in the intact, alert animal, we examined the response to hypoxia and reoxygenation in both control and CIH mice. Both groups exhibited qualitatively similar stereotypical responses to hypoxia (10% O_2_ inspired) and reoxygenation (Figure 6A). Although, no differences during the perihypoxic interval were found between the control and CIH groups in the overall pattern of minute ventilation (Figure 6B) or tidal volume (Figure 6C), a difference between groups was observed in respiratory frequency (Figure 6D). Comparisons specifically during air and hypoxia reveal, however, that CIH led to a decrease in frequency yet an increase in tidal volume (Table 2).

**Table 2:** Respiratory metrics recorded while breathing air or hypoxia.

		**Mass (g)**	**Tidal volume (μL × breath)**	**Respiratory frequency (breath × min^−1^)**	**Minute ventilation (μL × g^−1^ × min^−1^)**	***n***
Air^1^	Control	7.78 ± 0.2	27.3 ± 1.1	238 ± 17	6.5 ± 0.6	11
	CIH	7.97 ± 0.3	20.7 ± 2.5	306 ± 8	6.1 ± 0.6	12
	*P*-value	0.68	0.03	0.003	0.67	
Hypoxia^2^	Control	7.7 ± 0.2	19.1 ± 2.0	210 ± 11	7.4 ± 0.8	9
	CIH	7.9 ± 0.3	14.2 ± 1.2	256 ± 10	7.3 ± 0.9	11
	*P*-value	0.64	0.05	0.006	0.99	

*Respiratory metrics were measured during final minute in air prior to hypoxia and in the final minute of a 5 min exposure to hypoxia*.

1 *Air defined as inspired 21% O_2_ 0% CO_2_*.

2 *Hypoxia defined as inspired 10% O_2_ 0% CO_2_*.

## Discussion

Various forms of lesioning experiments indicate that the preBötzinger complex is essential for the generation of breathing (Ramirez et al., 1998; Gray et al., 2001; Tan et al., 2008). This network seems to be important for the generation of different types of inspiratory activities that are typically seen during different forms of oxygenation. While eupneic inspiratory activity is characteristic for the normoxic state, gasping activity is characteristic for the hypoxic state of the network. These different network states are characterized by different cellular and network properties (Hill et al., 2011; Garcia et al., 2013; Nieto-Posadas et al., 2014; Rivera-Angulo and Pena-Ortega, 2014). Indeed, the central respiratory network is very responsive to changes in oxygenation and undergoes a complex network reconfiguration as it transitions from a well-oxygenated to a hypoxic state. This reconfiguration is thought to contribute to the transition from eupnea to gasping and back from gasping to eupnea when changes in blood oxygenation occur *in vivo* (Lieske et al., 2000). Our results illustrate the ability of CIH to differentially influence the different states of the preBötC. Following CIH, rhythmogenesis from the preBötC appears to be more robust during hypoxia despite irregularities observed during baseline conditions. These observations support the notion that rhythmogenesis within the preBötC during baseline and hypoxic conditions represent fundamentally different network states that are differentially affected by CIH. Furthermore, along with other changes occurring throughout the nervous system, the effects of CIH during the perihypoxic interval may mechanistically contribute to the abnormal chemoreflex commonly found in individuals suffering from OSA (Deacon and Catcheside, 2015).

The state-dependency was mechanistically reflected in the strikingly different responses to the exposure to riluzole suggesting that CIH fundamentally alters the relative contribution of mechanisms underpinning rhythmogenesis. At the concentration used in the present study, neuronal sensitivity to riluzole in the preBötC appears to be due to the blockade of the persistent sodium current at the cellular level (I_NaP_; Pena et al., 2004; Ramirez et al., 2012), and at the network level, riluzole sensitivity is perhaps most evident during steady state hypoxia where I_NaP_ is a major contributor to the network rhythm (Pena et al., 2004; Ramirez et al., 2012). Indeed, riluzole inhibited hypoxic rhythmogenesis in both groups (*data not shown*), yet differences in riluzole sensitivity between control and CIH were evident during baseline conditions. Following the application of riluzole, baseline rhythmic activity became more regular (as evidenced by the IrS and sENT) in CIH exposed networks, while more irregular in control animals. Furthermore, during the transition to hypoxia and later during hypoxia, rhythmogenesis from the CIH group was also more robust when compared to control. Together these observations in riluzole and during hypoxia support the perspective that CIH may increase the contribution of I_NaP_ among preBötC neurons to affect rhythm generation both at baseline and hypoxic conditions. While hypoxia appears to reduce the strength of synaptic connectivity rather than eliminating functional connectivity in the preBötC (Nieto-Posadas et al., 2014), an enhanced I_NaP_ could strengthen functional connectivity throughout the network leading to the robust hypoxic rhythm generation observed following CIH. At the same time, an enhanced I_NaP_ could exaggerate the burst-to-burst irregularity under baseline conditions. Thus, blocking this current makes rhythm generation more regular.

A potentially enhanced I_NaP_ is not the only effect that CIH exerts on the mechanisms of rhythmogenesis. Indeed, following CIH, many preBötC neurons under baseline conditions exhibit reduced action potential generation during a network burst (Garcia et al., 2016), which may be the result of an increase in intrinsic or synaptic hyperpolarizing conductances. To this end, increased adrenergic tone combined with acute intermittent hypoxia has been shown to enhance glycinergic inhibition within the preBötC causing a long-lasting increase in irregularity (Zanella et al., 2014). As synaptic inhibition in the respiratory network is markedly reduced in hypoxia (Schmidt et al., 1995; Ramirez et al., 2012), such a phenomenon, may also occur with CIH exposure and contribute to the state dependent differences in the preBötC reported here. Moreover, CIH also increases heme-oxygenase 1 expression in the preBötC (Sunderram et al., 2016). Thus, the potential effect of CIH on the I_NaP_ is likely only one aspect of many changes and underscores the importance for continued investigation into the network and cellular level effects of CIH on the respiratory network.

The preBötC rhythm represents the output of phase-locking activity among non-pacemaker and pacemaker neurons with bursting properties (Carroll et al., 2013). The interaction among these neurons influence global network synchronization and can manifest as changes in the modulation of burst period and/or burst amplitude. We examined this modulation in various temporal windows using IrS to assess burst-to-burst variability and χ to assess the impact on the gain of inherent network fluctuations in a larger temporal window. In agreement with our recent work (Garcia et al., 2016), differences in baseline rhythmogenesis exist in the burst-to-burst window following CIH. Interestingly, this was not accompanied by changes to sENT suggesting that CIH does not affect the predictability of the overall pattern in the amplitude time series or the period time series. In simple terms, CIH increased the irregularity of amplitude and period not by rendering rhythm generation more unpredictable or “chaotic.” Instead, the respiratory rhythm generated by the preBötC fluctuates in amplitude and period over several cycles, and CIH increase the gain of these fluctuations. Interestingly, to the best of our knowledge, such periodic fluctuations in the generation of the inspiratory rhythm have not been described before—yet it is these baseline fluctuations that are most affected by exposure to CIH.

Interestingly, during hypoxia, the unequal variances between in IrS_AMP_ and IrS_P_ were not accompanied by effects on the gain of these inherent network fluctuations (i.e., χ) for the respective metrics when comparing CIH to control. Thus, when compared to rhythm generation during control conditions, CIH differentially affects hypoxic rhythmogenesis and suggests that the network rhythm during hypoxia represents a unique state distinct from that observed during control oxygen conditions.

In addition to determining how CIH affected the isolated preBötC, we examined how CIH exposure affected respiration during the peri-hypoxic interval. It has been previously reported that prior exposure to intermittent hypoxia suppressed gasping and autoresuscitation which is normally evoked during acute anoxia (0% O_2_ exposure, >20 min duration, Gozal et al., 2002). The suppression was dependent on the degree of hypoxia since a much different scenario occurs to the *in vivo* respiratory pattern when challenged with an acute bout of hypoxia (i.e., 10% O_2_ for 5 min, Peng et al., 2004). In our experimental conditions, we found that aerating aCSF with 0% O_2_ does not eliminate all oxygen in the media circulating to the brain slice (Garcia et al., 2010; Hill et al., 2011), which makes our conditions more similar to the situation studied by Peng et al. (2004). Indeed, we used also the same experimental paradigm as these authors.

Following CIH, rats exhibited an elevated respiratory frequency, tidal volume, and minute ventilation during the perihypoxic interval (Peng et al., 2004; Reeves and Gozal, 2006). While we also found that respiratory frequency increases, we did not observe a significant change in tidal volume. This discrepancy between studies may be due to species differences. Species differences in purinergic metabolism have been reported (Zwicker et al., 2011) and may be one of many potential differences that impact how CIH differentially respiratory metrics of the mouse and rat.

The effects of CIH respiratory activity has been largely attributed to CIH-mediated changes to peripheral input from the carotid bodies (Peng et al., 2004), the primary oxygen sensing organ in the body. However, carotid bodies must exert their effects on minute ventilation and amplitude by acting through the central rhythm-generating network. Thus, in considering how the carotid bodies will affect the ventilatory response one must also take into consideration that this central circuitry is also altered by CIH. It is difficult to predict how the changes in the preBötC influence the transmission of the peripheral chemoreceptors on the overall ventilatory output. The increased fluctuation in amplitude and frequency values suggests that CIH increased the gain of the centrally generated respiratory network rhythm, which could exaggerate an increased gain observed at the level of the peripheral chemoreceptors. The conclusions are further complicated by our finding that the effects of CIH on the centrally generated inspiratory rhythm were dependent on the state of oxygenation—exaggerating irregularities during well-oxygenated states yet facilitating rhythm generation during hypoxia. This could also introduce instabilities, if the network fluctuates between a normoxic and hypoxic state as is typical for obstructive sleep apnea. An increased gain in the peripheral as well as central nervous system would then exaggerate these instabilities (Peng et al., 2004), Clearly, it is difficult if not impossible to experimentally test or mechanistically dissect the relative contribution of the peripheral and central nervous system components toward the overall ventilatory response of the whole organism. But, what can be concluded is, that CIH affects not only the peripheral chemoreceptors but also alters the performance of central inspiratory rhythm generation in an oxygen-dependent manner. These state-dependent effects, in conjunction with the impact of CIH in the carotid bodies, could perpetuate instability and the occurrence of apnea by increasing the responsiveness of ventilation to deviations in blood gases.

## Author contributions

Conceived and designed the experiments: AG, and JR. Performed the experiments: AG, TD, and MK. Analyzed the data: AG, TD, and MK. Contributed reagents/materials/analysis tools: JR. Wrote the paper: AG and JR.

### Conflict of interest statement

The authors declare that the research was conducted in the absence of any commercial or financial relationships that could be construed as a potential conflict of interest.

## References

[B1] Ballanyi K. Ruangkittisakul A. (2009). Structure-function analysis of rhythmogenic inspiratory pre-Botzinger complex networks in “calibrated” newborn rat brainstem slices. Respir. Physiol. Neurobiol. 168, 158–178. 10.1016/j.resp.2009.04.020 19406253

[B2] Blitz D. M. Ramirez J. M. (2002). Long-term modulation of respiratory network activity following anoxia *in vitro*. J. Neurophysiol. 87, 2964–2971. 10.1152/jn.00515.2001 12037199

[B3] Carroll M. S. Viemari J. C. Ramirez J. M. (2013). Patterns of inspiratory phase-dependent activity in the *in vitro* respiratory network. J. Neurophysiol. 109, 285–295. 10.1152/jn.00619.2012 23076109 PMC3545453

[B4] Chopra S. Polotsky V. Y. Jun J. C. (2016). Sleep apnea research in animals. Past, Present, and Future. Am. J. Respir. Cell Mol. Biol. 54, 299–305. 10.1165/rcmb.2015-0218TR 26448201 PMC4821036

[B5] Davies C. R. Harrington J. J. (2016). Impact of obstructive sleep apnea on neurocognitive function and impact of continuous positive air pressure. Sleep Med. Clin. 11, 287–298. 10.1016/j.jsmc.2016.04.006 27542875

[B6] Deacon N. L. Catcheside P. G. (2015). The role of high loop gain induced by intermittent hypoxia in the pathophysiology of obstructive sleep apnoea. Sleep Med. Rev. 22, 3–14. 10.1016/j.smrv.2014.10.003 25454671

[B7] Del Negro C. A. Morgado-Valle C. Feldman J. L. (2002). Respiratory rhythm: an emergent network property? Neuron 34, 821–830. 10.1016/S0896-6273(02)00712-2 12062027

[B8] Dempsey J. A. Veasey S. C. Morgan B. J. O'donnell C. P. (2010). Pathophysiology of sleep apnea. Physiol. Rev. 90, 47–112. 10.1152/physrev.00043.2008 20086074 PMC3970937

[B9] Devinney M. J. Nichols N. L. Mitchell G. S. (2016). Sustained hypoxia elicits competing spinal mechanisms of phrenic motor facilitation. J. Neurosci. 36, 7877–7885. 10.1523/JNEUROSCI.4122-15.2016 27466333 PMC4961775

[B10] Di Fiore J. M. Poets C. F. Gauda E. Martin R. J. Macfarlane P. (2016). Cardiorespiratory events in preterm infants: interventions and consequences. J. Perinatol. 36, 251–258. 10.1038/jp.2015.165 26583943

[B11] Garcia A. J. III. Putnam R. W. Dean J. B. (2010). Hyperbaric hyperoxia and normobaric reoxygenation increase excitability and activate oxygen-induced potentiation in CA1 hippocampal neurons. J. Appl. Physiol. (1985) 109, 804–819. 10.1152/japplphysiol.91429.2008 20558753 PMC2944633

[B12] Garcia A. J. III. Rotem-Kohavi N. Doi A. Ramirez J. M. (2013). Post-hypoxic recovery of respiratory rhythm generation is gender dependent. PLoS ONE 8:e60695. 10.1371/journal.pone.0060695 23593283 PMC3620234

[B13] Garcia A. J. III. Zanella S. Dashevskiy T. Khan S. A. Khuu M. A. Prabhakar N. R.. (2016). Chronic intermittent hypoxia alters local respiratory circuit function at the level of the prebotzinger complex. Front. Neurosci. 10:4. 10.3389/fnins.2016.00004 26869872 PMC4740384

[B14] Gozal D. Gozal E. Reeves S. R. Lipton A. J. (2002). Gasping and autoresuscitation in the developing rat: effect of antecedent intermittent hypoxia. J. Appl. Physiol. (1985) 92, 1141–1144. 10.1152/japplphysiol.00972.2001 11842051

[B15] Gozal D. Kheirandish-Gozal L. Bhattacharjee R. Spruyt K. (2010). Neurocognitive and endothelial dysfunction in children with obstructive sleep apnea. Pediatrics 126, e1161–e1167. 10.1542/peds.2010-0688 20956420

[B16] Gray P. A. Janczewski W. A. Mellen N. McCrimmon D. R. Feldman J. L. (2001). Normal breathing requires preBotzinger complex neurokinin-1 receptor-expressing neurons. Nat. Neurosci. 4, 927–930. 10.1038/nn0901-927 11528424 PMC2810393

[B17] Hill A. A. Garcia A. J. III. Zanella S. Upadhyaya R. Ramirez J. M. (2011). Graded reductions in oxygenation evoke graded reconfiguration of the isolated respiratory network. J. Neurophysiol. 105, 625–639. 10.1152/jn.00237.2010 21084689 PMC3059168

[B18] Javaheri S. Barbe F. Campos-Rodriguez F. Dempsey J. A. Khayat R. Javaheri S.. (2017). Sleep apnea: types, mechanisms, and clinical cardiovascular consequences. J. Am. Coll. Cardiol. 69, 841–858. 10.1016/j.jacc.2016.11.069 28209226 PMC5393905

[B19] Lieske S. P. Ramirez J. M. (2006a). Pattern-specific synaptic mechanisms in a multifunctional network. I. Effects of alterations in synapse strength. J. Neurophysiol. 95, 1323–1333. 10.1152/jn.00505.2004 16492944

[B20] Lieske S. P. Ramirez J. M. (2006b). Pattern-specific synaptic mechanisms in a multifunctional network. II. Intrinsic modulation by metabotropic glutamate receptors. J. Neurophysiol. 95, 1334–1344. 10.1152/jn.00506.2004 16492945

[B21] Lieske S. P. Thoby-Brisson M. Telgkamp P. Ramirez J. M. (2000). Reconfiguration of the neural network controlling multiple breathing patterns: eupnea, sighs and gasps [see comment]. Nat. Neurosci. 3, 600–607. 10.1038/75776 10816317

[B22] Malhotra A. White D. P. (2002). Obstructive sleep apnoea. Lancet 360, 237–245. 10.1016/S0140-6736(02)09464-3 12133673

[B23] Navarrete-Opazo A. Mitchell G. S. (2014). Therapeutic potential of intermittent hypoxia: a matter of dose. Am. J. Physiol. Regul. Integr. Comp. Physiol. 307, R1181–R1197. 10.1152/ajpregu.00208.2014 25231353 PMC4315448

[B24] Nichols N. L. Dale E. A. Mitchell G. S. (2012). Severe acute intermittent hypoxia elicits phrenic long-term facilitation by a novel adenosine-dependent mechanism. J. Appl. Physiol. (1985) 112, 1678–1688. 10.1152/japplphysiol.00060.2012 22403346 PMC3365407

[B25] Nieto-Posadas A. Flores-Martinez E. Lorea-Hernandez J. J. Rivera-Angulo A. J. Perez-Ortega J. E. Bargas J.. (2014). Change in network connectivity during fictive-gasping generation in hypoxia: prevention by a metabolic intermediate. Front. Physiol. 5:265. 10.3389/fphys.2014.00265 25101002 PMC4107943

[B26] Pena F. Parkis M. A. Tryba A. K. Ramirez J. M. (2004). Differential contribution of pacemaker properties to the generation of respiratory rhythms during normoxia and hypoxia. Neuron 43, 105–117. 10.1016/j.neuron.2004.06.023 15233921

[B27] Peng Y. J. Nanduri J. Yuan G. Wang N. Deneris E. Pendyala S.. (2009). NADPH oxidase is required for the sensory plasticity of the carotid body by chronic intermittent hypoxia. J. Neurosci. 29, 4903–4910. 10.1523/JNEUROSCI.4768-08.2009 19369559 PMC2692682

[B28] Peng Y. J. Rennison J. Prabhakar N. R. (2004). Intermittent hypoxia augments carotid body and ventilatory response to hypoxia in neonatal rat pups. J. Appl. Physiol. (1985) 97, 2020–2025. 10.1152/japplphysiol.00876.2003 15258129

[B29] Quintana P. Soto D. Poirot O. Zonouzi M. Kellenberger S. Muller D.. (2015). Acid-sensing ion channel 1a drives AMPA receptor plasticity following ischaemia and acidosis in hippocampal CA1 neurons. J. Physiol. 593, 4373–4386. 10.1113/JP270701 26174503 PMC4594240

[B30] Ramirez J. M. Doi A. Garcia A. J. III. Elsen F. P. Koch H. Wei A. D. (2012). The cellular building blocks of breathing. Compr. Physiol. 2, 2683–2731. 10.1002/cphy.c110033 23720262 PMC3684023

[B31] Ramirez J. M. Garcia A. J. III. Anderson T. M. Koschnitzky J. E. Peng Y. J. Kumar G. K.. (2013). Central and peripheral factors contributing to obstructive sleep apneas. Respir. Physiol. Neurobiol. 189, 344–353. 10.1016/j.resp.2013.06.004 23770311 PMC3901437

[B32] Ramirez J. M. Schwarzacher S. W. Pierrefiche O. Olivera B. M. Richter D. W. (1998). Selective lesioning of the cat pre-Botzinger complex *in vivo* eliminates breathing but not gasping. J. Physiol. 507(Pt 3), 895–907. 10.1111/j.1469-7793.1998.895bs.x 9508848 PMC2230836

[B33] Reeves S. R. Gozal D. (2006). Respiratory and metabolic responses to early postnatal chronic intermittent hypoxia and sustained hypoxia in the developing rat. Pediatr. Res. 60, 680–686. 10.1203/01.pdr.0000246073.95911.18 17065578

[B34] Richman J. S. Moorman J. R. (2000). Physiological time-series analysis using approximate entropy and sample entropy. Am. J. Physiol. Heart Circ. Physiol. 278, H2039–H2049. Available online at: http://ajpheart.physiology.org/content/278/6/H2039.long 10843903 10.1152/ajpheart.2000.278.6.H2039

[B35] Rivera-Angulo A. J. Pena-Ortega F. (2014). Isocitrate supplementation promotes breathing generation, gasping, and autoresuscitation in neonatal mice. J. Neurosci. Res. 92, 375–388. 10.1002/jnr.23330 24375766

[B36] Rybak I. A. Abdala A. P. Markin S. N. Paton J. F. Smith J. C. (2007). Spatial organization and state-dependent mechanisms for respiratory rhythm and pattern generation. Prog. Brain Res. 165, 201–220. 10.1016/S0079-6123(06)65013-9 17925248 PMC2408750

[B37] Schmidt C. Bellingham M. C. Richter D. W. (1995). Adenosinergic modulation of respiratory neurones and hypoxic responses in the anaesthetized cat. J. Physiol. 483(Pt 3), 769–781. 10.1113/jphysiol.1995.sp020621 7776257 PMC1157817

[B38] Sunderram J. Semmlow J. Patel P. Rao H. Chun G. Agarwala P.. (2016). Heme oxygenase-1-dependent central cardiorespiratory adaptations to chronic intermittent hypoxia in mice. J. Appl. Physiol. (1985) 121, 944–952. 10.1152/japplphysiol.00036.2016 27609199

[B39] Tan W. Janczewski W. A. Yang P. Shao X. M. Callaway E. M. Feldman J. L. (2008). Silencing preBotzinger complex somatostatin-expressing neurons induces persistent apnea in awake rat. Nat. Neurosci. 11, 538–540. 10.1038/nn.2104 18391943 PMC2515565

[B40] Telgkamp P. Cao Y. Q. Basbaum A. I. Ramirez J. M. (2002). Long-term deprivation of substance P in PPT-A mutant mice alters the anoxic response of the isolated respiratory network. J. Neurophysiol. 88, 206–213. 10.1152/jn.00676.2001 12091546

[B41] Tryba A. K. Pena F. Lieske S. P. Viemari J. C. Thoby-Brisson M. Ramirez J. M. (2008). Differential modulation of neural network and pacemaker activity underlying eupnea and sigh-breathing activities. J. Neurophysiol. 99, 2114–2125. 10.1152/jn.01192.2007 18287547 PMC3860370

[B42] Viemari J. C. Garcia A. J. III. Doi A. Elsen G. Ramirez J. M. (2013). β-Noradrenergic receptor activation specifically modulates the generation of sighs *in vivo* and *in vitro*. Front. Neural Circuits 7:179. 10.3389/fncir.2013.00179 24273495 PMC3824105

[B43] Zanella S. Doi A. Garcia A. J. III. Elsen F. Kirsch S. Wei A. D.. (2014). When norepinephrine becomes a driver of breathing irregularities: how intermittent hypoxia fundamentally alters the modulatory response of the respiratory network. J. Neurosci. 34, 36–50. 10.1523/JNEUROSCI.3644-12.2014 24381266 PMC3866492

[B44] Zwicker J. D. Rajani V. Hahn L. B. Funk G. D. (2011). Purinergic modulation of preBotzinger complex inspiratory rhythm in rodents: the interaction between ATP and adenosine. J. Physiol. 589, 4583–4600. 10.1113/jphysiol.2011.210930 21788352 PMC3208226

